# *TMPRSS2‐ERG* fusions linked to prostate cancer racial health disparities: A focus on Africa

**DOI:** 10.1002/pros.23823

**Published:** 2019-05-15

**Authors:** James Blackburn, Stefano Vecchiarelli, Erin E. Heyer, Sean M. Patrick, Ruth J. Lyons, Weerachai Jaratlerdsiri, Smit van Zyl, M. S. Riana Bornman, Tim R. Mercer, Vanessa M. Hayes

**Affiliations:** ^1^ Garvan Institute of Medical Research The Kinghorn Cancer Centre Darlinghurst Australia; ^2^ St. Vincent's Clinical School Faculty of Medicine, UNSW Sydney Australia; ^3^ School of Health Systems and Public Health University of Pretoria Gezina South Africa; ^4^ University of Limpopo, Turfloop Campus Sovenga South Africa; ^5^ Altius Institute for Biomedical Sciences Seattle Washington; ^6^ Sydney Medical School University of Sydney Camperdown Australia

**Keywords:** African ancestry, early‐onset, fusion gene, prostate cancer, racial health disparity, *TMPRSS2*‐ *ERG*

## Abstract

**Background:**

The androgen‐regulated gene *TMPRSS2* to the ETS transcription factor gene *ERG* fusion is the most common genomic alteration acquired during prostate tumorigenesis and biased toward men of European ancestry. In contrast, African American men present with more advanced disease, yet their tumors are less likely to acquire *TMPRSS2‐ERG*. Data for Africa is scarce.

**Methods:**

RNA was made available for genomic analyses from 181 prostate tissue biopsy cores from Black South African men, 94 with and 87 without pathological evidence for prostate cancer. Reverse transcription polymerase chain reaction was used to screen for the *TMPRSS2‐ERG* fusion, while transcript junction coordinates and isoform frequencies, including novel gene fusions, were determined using targeted RNA sequencing.

**Results:**

Here we report a frequency of 13% for *TMPRSS2‐ERG* in tumors from Black South Africans. Present in 12/94 positive versus 1/87 cancer negative prostate tissue cores, this suggests a 92.62% predictivity for a positive cancer diagnosis (*P *=* *0.0031). At a frequency of almost half that reported for African Americans and roughly a quarter of that reported for men of European ancestry, acquisition of *TMPRSS2‐ERG* appears to be inversely associated with aggressive prostate cancer. Further support was provided by linking the presence of *TMPRSS2‐ERG* to low‐grade disease in younger patients (*P *=* *0.0466), with higher expressing distal *ERG* fusion junction coordinates.

**Conclusions:**

Only the second study of its kind for the African continent, we support a link between *TMPRSS2‐ERG* status and prostate cancer racial health disparity beyond the borders of the United States. We call for urgent evaluation of androgen deprivation therapy within Africa.

## 1 INTRODUCTION

The most frequent somatic alteration in prostate cancer (PCa) involves an intrachromosomal translocation or deletion event on the long arm of chromosome 21. This genomic alteration appears to occur early in tumourigenesis, resulting in fusion of the androgen‐regulated transmembrane protease serine 2 gene (*TMPRSS2*) to a member of the transcription factor erythroblastosis virus E26 transforming sequence family (*ERG*)^1^, leading to androgen‐dependent overexpression of *ERG.* 2 The frequency of *TMPRSS2‐ERG* fusions in PCa shows a notable racial disparity. A recent meta‐analysis confirmed the highest prevalence rates in men of European ancestry (49%), with lower levels reported for men of Asian (27%) and African (25%) ancestries.^3^ The latter includes three African American studies (73/260; 28%) and a single study from West Africa, specifically Ghana (47/262; 18%). There also appears to be racial discordance in the mechanism of *TMPRSS2‐ERG* occurrence, with African Americans most commonly exhibiting fusion through deletion, and European and Asian Americans through translocation.^4^ Intriguingly, African ancestry is significantly associated with high‐grade (Gleason score ≥ 8) PCa,^5^ which questions the role of *TMPRSS2‐ERG* fusions in tumor development in African populations. Reporting a 2.1‐fold increase in high‐grade PCa presentation in Black South African men compared with African Americans,^6^ we sought to determine the frequency, composition, and clinicopathological association of acquired *TMPRSS2‐ERG* fusions within non‐American men of African ancestry.

## 2 PATIENTS AND METHODS

### 2.1 Clinicopathological overview and ethics

Patients were enrolled at time of diagnosis as part of the Southern African Prostate Cancer Study (SAPCS)^6^ and presented at one of the participating urological clinics within the Provinces of Limpopo or Gauteng of South Africa. All self‐identified ethnically as Black South African, including a single Black Zimbabwean, from one of 11 ethnic groupings, with age and prostate specific antigen levels recorded at time of diagnosis (Table 1). Clinicopathological analyses confirmed 94 men with PCa (positive Gleason score) and 87 without PCa (negative Gleason score), either with (*n* = 56) or without (*n* = 31) the presence of benign prostate hyperplasia.

**Table 1:** Clinical and pathological characteristics

		PCa	BPH	No PCa
Number of patients (*n* = 181)		94	56	31
Ethnicity	Pedi	39	27	22
	Sotho	5	1	2
	Swazi	2	3	2
	Tsonga	8	6	1
	Tswana	3	4	0
	Venda	8	6	2
	Ndebele	18	5	0
	Zulu	6	3	2
	Xhosa	3	0	0
	Colored	1	1	0
	Shona	1	0	0
Median (IQR) age at diagnosis in years		69 (44‐94)	69 (53‐89)	67 (50‐92)
Median (IQR) PSA at diagnosis ng/mL		51.5 (4.4‐2000)	17.3 (2.2‐1000)	16.1 (2‐1475)
Pathological grading	ISUP1	18	–	–
	ISUP2	18	–	–
	ISUP3	12	–	–
	ISUP4	23	–	–
	ISUP5	23	–	–

Abbreviations: BPH, benign prostate hyperplasia; IQR, interquartile range; ISUP, International Society of Urological Pathology grading system; PCa, prostate cancer; PSA, prostate specific antigen.

The study was approved by the Medical/Human Research Ethics Committee's (MREC or HREC) from the University of Limpopo's Medunsa Campus (MREC/H/28/2009) and the University of Pretoria's Faculty of Health Sciences (HREC #43/2010) in South Africa. Patients provided written informed consent before enrollment. Prostate tissue samples were shipped to the Garvan Institute of Medical Research under the Republic of South Africa Department of Health Export Permit, in accordance with the National Health Act 2003 (J1/2/4/2 #1/12). Analysis of the samples was performed in accordance with St Vincent's Hospital (SVH) HREC site‐specific approval (#SVH15/227).

### 2.2 TMPRSS2‐ERG screening

**Figure 1:**
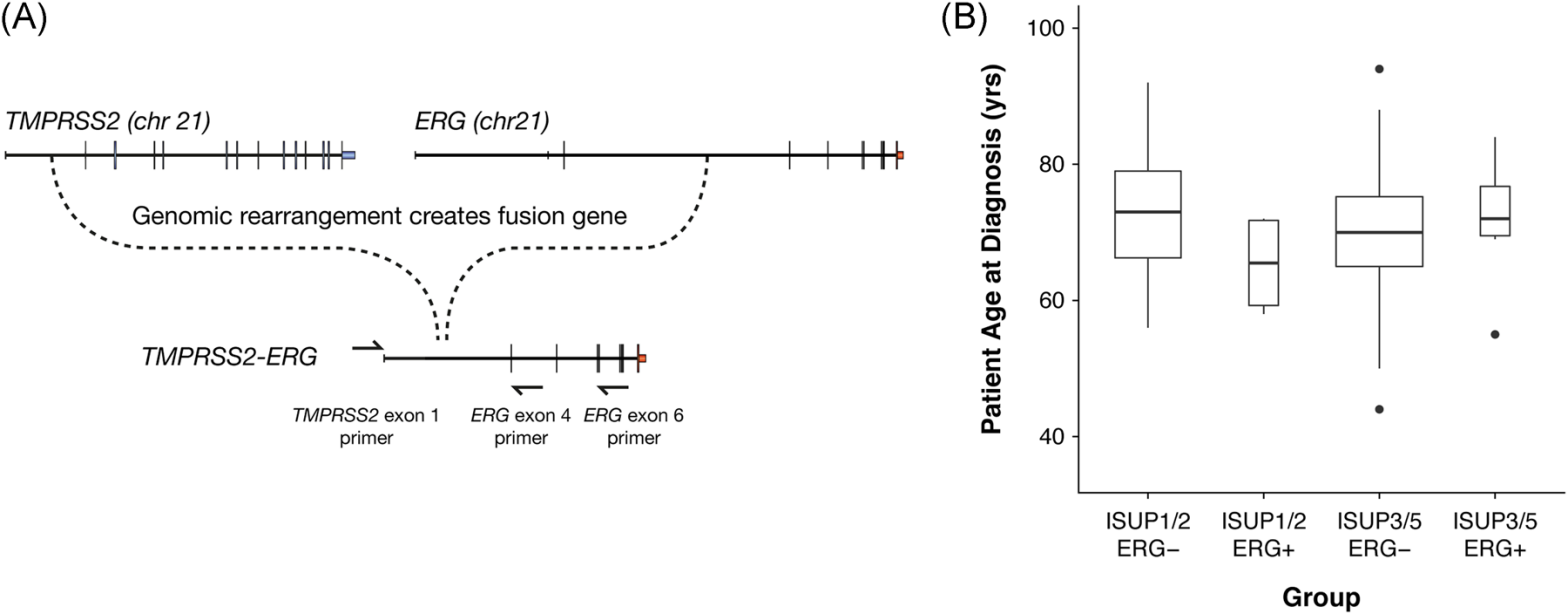
Summary of *TMPRSS2‐ERG* fusion gene diagnosis. (A) Schematic representation of the fusion gene resulting from a genomic rearrangement within *TMPRSS2* intron 1 and *ERG* intron 3. Locations of forward *TMPRSS2* and reverse *ERG* PCR primers are shown as arrows. (B) Difference in age at diagnosis for *TMPRSS2*‐*ERG* positive versus negative low‐grade (ISUP 1 and 2) and high‐grade (ISUP 3 to 5) tumors

RNA was prepared from 181 prostate tissue core biopsies. Presence of the *TMPRSS2‐ERG* fusion gene was determined using two sets of previously described reverse transcription polymerase chain reaction (RT‐PCR) primer pairs,^1^ including a single forward primer located in exon 1 of *TMPRSS2*: 5′‐CAGGAGGCGGAGGCGGA‐3′ and two *ERG* reverse primers located in exon 4: 5′‐GTAGGCACACTCAAACAACGACTGG‐3′ and exon 6: 5′‐GGCGTTGTAGCTGGGGGTGAG‐3′ (Figure 1A). PC3 and VCaP cell lines represented *TMPRSS2*‐*ERG* fusion gene negative and positive controls, respectively.

### 2.3 Targeted fusion RNA sequencing

Targeted RNA sequencing (RNA CaptureSeq) was used to generate a snapshot validation for RT‐PCR screening and to define the exact *TMPRSS2‐ERG* fusion transcript junction coordinates. In brief, sequencing libraries were prepared from 1 μg of patient RNA using the KAPA Stranded RNA‐Seq Library Preparation Kit (Roche, Basel, Switzerland). Capture was performed according to manufacturer's instructions (Roche) using custom biotinylated probes including known PCa associated chromosomal translocations. Captured libraries were sequenced to standard depth (125‐bp paired‐end) using the Illumina HiSeq. 2500 System v4 (Illumina, San Diego, CA). Post‐filtering, reads were mapped to human reference genome hg38 with STAR v2.4.2a^7^ and fusion genes identified with STAR‐Fusion and FusionCatcher v0.99.6 beta.^8^ Only the fusion transcript isoforms identified by both programs within each patient sample were used in further analysis.

### 2.4 TMPRSS2‐ERG expression

Expression of the *TMPRSS2‐ERG* fusion gene in each patient was calculated by normalizing the total number of fusion supporting reads to individual sequencing library sizes.

### 2.5 Statistics

Variables between groups were analyzed using Fisher's exact test. *P* < 0.05 were considered statistically significant.

## 3 RESULTS

### 3.1 Frequency and distribution of *TMPRSS2‐ERG* fusion

The frequency of *TMPRSS2*‐*ERG* fusion gene occurrence was 12.8% (12/94) in PCa and 1.5% (1/87) in noncancerous prostate tissue. Thus the presence of *TMPRSS2*‐*ERG* in this population is 92.62% predictive for a positive diagnosis of PCa (*P* = 0.0031), with a negative predictive value of 51.25%.

Although rare, we observed an even distribution for *TMPRSS2*‐*ERG* in patients defined at clinical presentation by the International Society of Urological Pathology (ISUP)^9^ as high‐grade (ISUP 3 to 5; Gleason score ≥ 4 + 3; *n* = 6) or low‐grade (ISUP 1 and 2; Gleason score ≤ 3 + 4; *n* = 6) (Table 1). We found the median age at diagnosis for patients presenting with low‐grade *TMPRSS2*‐*ERG* positive tumors (65.5 years) was almost 8 years younger than patients with low‐grade *TMPRSS2*‐*ERG* negative tumors (73 years, *P *=* *0.04656) and almost 7 years younger than *TMPRSS2*‐*ERG* positive high‐grade tumors (72 years) (Figure 1B).

### 3.2 *TMPRSS2‐ERG* validation, isoforms and ERG expression

Using our targeted RNA CaptureSeq method, we validated the RT‐PCR screening approach confirming *TMPRSS2*‐*ERG* status for the 12 fusion positive and 12 randomly selected early‐onset (range 44 to 62 years) fusion negative PCa cases. Each *TMPRSS2‐ERG* fusion positive tumor presented with minimally two and maximally five *TMPRSS2‐ERG* fusion isoforms (total of 40 fusion junctions), with three patients also presenting a novel fusion event: *RP11‐356O9.1‐ETV1* in two patients and *PTPRK‐LAMA2* in a single patient. No novel fusion events were identified within the fusion negative tumors.

**Figure 2:**
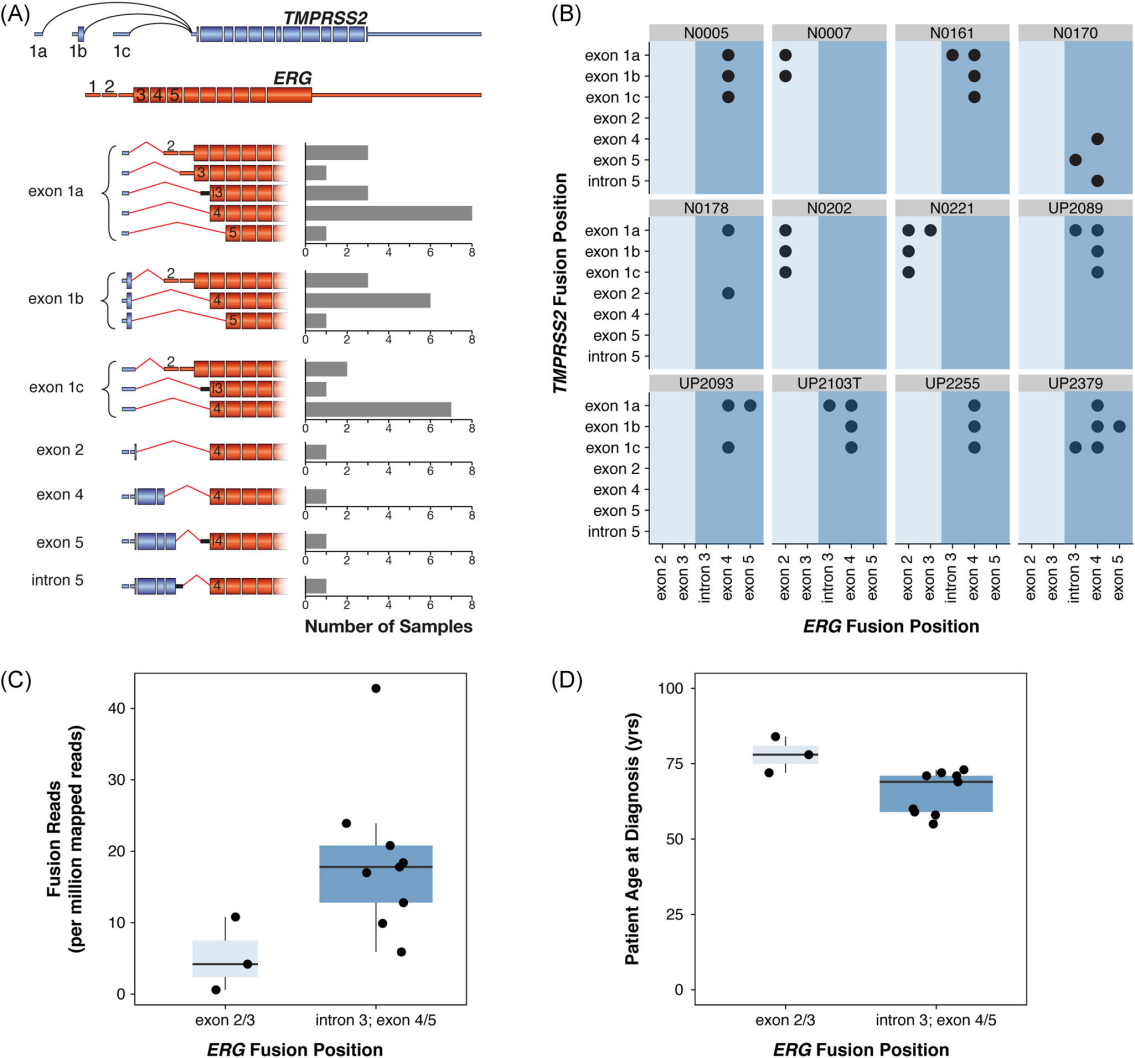
*TMPRSS2*‐*ERG* fusion gene isoforms detected in 12 prostate tumors from African patients. (A) *TMPRSS2* and *ERG* gene structures and *TMPRSS2*‐*ERG* fusion isoform prevalence across all patients based on the GENCODE v27 comprehensive exon annotation. Bar charts quantify the number of samples expressing each isoform. For simplicity, *TMPRSS2* junctions beyond exon 1 are depicted with exon 1a. Black line represents retained intronic sequence. (B) Isoform prevalence, represented as fusion position between *TMPRSS2* and *ERG* for each patient. Light and dark blue panels denote proximal and distal *ERG* junction positions. (C) Boxplot of *TMPRSS2*‐*ERG* expression relative to ERG fusion position. (D) Boxplot of patient age at diagnosis relative to ERG fusion position. Each dot represents a single patient sample [Color figure can be viewed at wileyonlinelibrary.com]

Consistent with previous findings, the most common *TMPRSS2* fusion junctions were located within the first intron downstream of three alternative noncoding exons, with only two samples utilizing *TMPRSS2* fusion coordinates beyond exon 2 (4/40) (Figure 2A). Similarly, *ERG* fusion junctions were dominated by a fusion upstream of exon 4 (24/40), with the second most common being a fusion upstream of exon 2 (8/40, traditionally noncoding). Within each patient tumor, *ERG* fusion coordinates always occurred either upstream or downstream of exon 3, but never both (Figure 2B).

Though not statistically significant due to small sample size, there was a striking difference in the expression levels of patients presenting with either the proximal or distal *ERG* fusion coordinates, with higher expression detected from the distal isoforms (Figure 2C). Additionally, patients possessing the higher expressing distal *ERG* fusion coordinates presented earlier at diagnosis, with a median age of 69 years (range 55 to 73 years) compared with the three patients with the lower expressing proximal coordinates, at 72, 78, and 81 years (Figure 2D).

## 4 DISCUSSION

Although genomic instability is assumed to increase with tumor progression,^10^ we show that Black South African men with significant high‐grade PCa have lower frequencies of *TMPRSS2*‐*ERG* fusion than men of European ancestry. When present, we show a tendency for association with early‐onset low‐grade PCa presentation, with higher expression from distal *ERG* junction coordinates. Experimental data performed in prostate epithelial cells supports the hypothesis that increased androgen levels in younger men may be directly related to the increase in *TMPRSS2*‐*ERG* expression,^11^ while a previous European‐based study reported accumulation of nonandrogen associated genomic alterations with patient and tumor age, dependent on *TMPRSS2*‐*ERG* status.^12^

Interestingly, we identified two novel fusion events including *ETV1*—commonly fused in PCa—with novel partner *RP11‐356O9.1* (in 2/12 *TMPRSS2*‐*ERG* positive tumors), and the well‐known colorectal fusion gene *PTPRK* 13 fused with novel partner *LAMA2.* While no novel fusion events were identified in the 12 *TMPRSS2*‐*ERG* negative tumors using our targeted RNA CaptureSeq method, through the first deep whole genome sequencing of tumor‐normal pairs from six African high‐risk treatment‐naïve PCa patients, we recently reported a significant increase in small somatic variation (tumor mutational burden; 3.8 mutations/Mbp), lack of *ETS* fusions and under‐representation of complex genomic rearrangements, compared with European‐derived matched tumors.^14^ As such, we have recently called for much needed genome profiling of PCa within African populations to help explain the apparent racial health disparities.^15^

While androgen deprivation therapy (ADT) administered through hormone blockers are traditionally used for treating advanced PCa, in Southern Africa orchidectomy is still common practice. Either way, the goal is to inhibit androgen receptor (AR) signaling.^16^ Combining ADT with docetaxel, a taxane chemotherapy, has been shown to provide further survival benefit to patients.^17^ The positive effect of docetaxel is believed to act, at least in part, through impairing AR activity.^18^ In turn, *TMPRSS2‐ERG* fusion positive patients appear to respond well to ADT and neoadjuvant docetaxel, compared with untreated patients.^19^ More recently, a large study out of China suggests that *TMPRSS2‐ERG* fusion is a biomarker for predicting response to ADT.^20^ The lack of androgen‐regulated *TMPRSS2‐ERG* fusion within African populations biased toward aggressive disease presentation, calls for immediate assessment of the true benefit of ADT, taking into consideration significant long‐term side effects of the more commonly practiced orchidectomy, within the continent.

## 5 SUMMARY

To the best of our knowledge, this is the second only PCa study of its kind for Africa and the first for southern Africa. Built on observations made within African Americans and combined with the single study from Ghana, it is becoming evident that *TMPRSS2‐ERG* fusions are significantly less common in driving prostate tumourigenesis within populations from Africa. This is further supported by an inverse association between presence and adverse outcomes, as well as our observed link with younger‐onset low‐risk disease.

## CONFLICT OF INTERESTS

The authors declare that there are no conflict of interests.

## AUTHOR CONTRIBUTION

VMH, JB, and TRM designed the study. SMP, SvZ, and MSRB consented, performed clinical evaluation, and processed the samples. JB, SV, and RJL performed the experiments. JB, EEH, WJ, and VMH performed analysis and interpretation of results. JB and VMH drafted the manuscript. All authors reviewed the manuscript.
